# Structure of neprilysin in complex with the active metabolite of sacubitril

**DOI:** 10.1038/srep27909

**Published:** 2016-06-15

**Authors:** Nikolaus Schiering, Allan D’Arcy, Frederic Villard, Paul Ramage, Claude Logel, Frederic Cumin, Gary M. Ksander, Christian Wiesmann, Rajeshri G. Karki, Muneto Mogi

**Affiliations:** 1 Novartis Institutes for BioMedical Research Inc., Fabrikstrasse 16, CH-4002 Basel, Switzerland; 2 Novartis Institutes for BioMedical Research Inc., 100 Technology Square, Cambridge, Massachusetts, 02139, United States

## Abstract

Sacubitril is an ethyl ester prodrug of LBQ657, the active neprilysin (NEP) inhibitor, and a component of LCZ696 (sacubitril/valsartan). We report herein the three-dimensional structure of LBQ657 in complex with human NEP at 2 Å resolution. The crystal structure unravels the binding mode of the compound occupying the S1, S1’ and S2’ sub-pockets of the active site, consistent with a competitive inhibition mode. An induced fit conformational change upon binding of the P1’-biphenyl moiety of the inhibitor suggests an explanation for its selectivity against structurally homologous zinc metallopeptidases.

Sacubitril/valsartan (LCZ696) was recently approved by the European Commission and the U.S. Food and Drug Administration to reduce the risk of cardiovascular death in heart failure patients with reduced ejection fraction^1^. Nearly 6 million people in the US suffer from heart failure and about half have the reduced ejection fraction form^2^. LCZ696 (Fig. 1) is a sodium salt complex comprising the anionic forms of the angiotensin II type-1 receptor antagonist valsartan and the NEP inhibitor prodrug sacubitril^3^. Upon oral administration, LCZ696 delivers rapid systemic exposure to sacubitril and valsartan. Sacubitril is converted by enzymatic cleavage of the ethyl ester to the active metabolite LBQ657^4^. LBQ657 is a single diastereomer with specific stereocenters (Fig. 1).

Neprilysin, also called neutral endopeptidase, enkephalinase, or atriopeptidase, is a zinc dependent type II integral membrane peptidase belonging to the M13 family. It degrades a variety of vasoactive peptides such as atrial natriuretic peptide, brain natriuretic peptide, bradykinin, adrenomedullin and endothelin-1^5^. NEP inhibition leads to an increased level of these peptides. The combination of NEP and angiotensin receptor inhibition is superior to either agent alone and leads to vasodilation and reduction of extracellular fluid via sodium excretion^6^.

In recent years, several structures of the soluble extracellular domain of NEP complexed with inhibitors have become available^7^,^8^,^9^,^10^,^11^. In this report we describe the crystal structure of NEP in complex with LBQ657 and rationalize the selectivity of the compound relative to the homologous peptidases, endothelin converting enzyme (ECE-1) and neprilysin 2 (NEP2).

## Results and Discussion

**Figure 2:**
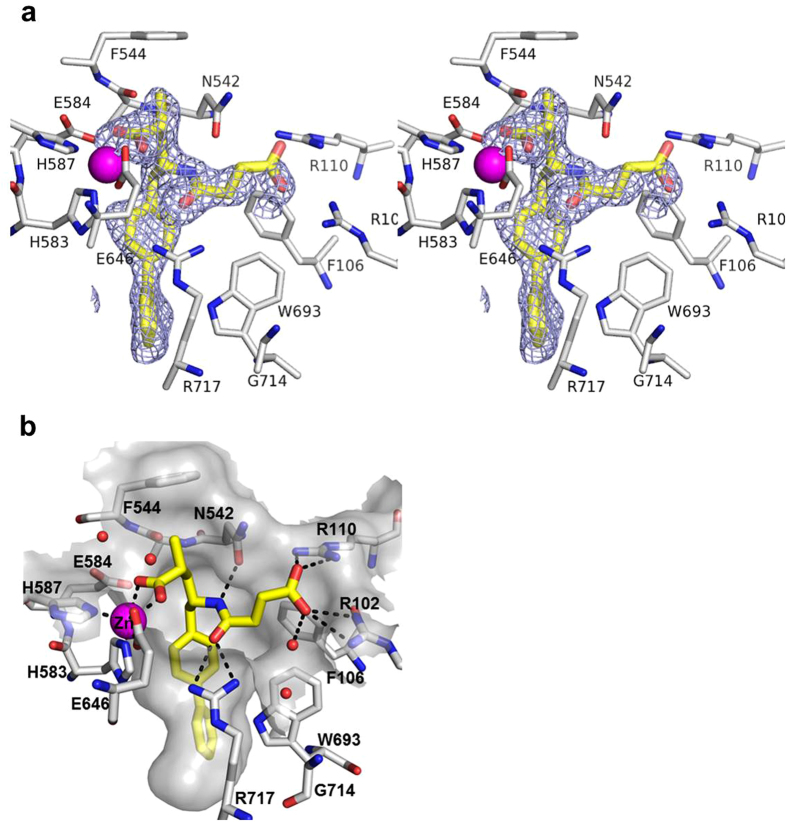
Active site view of NEP in complex with LBQ657. (**a**) Initial Fo-Fc difference electron density map for LBQ657 is shown in blue mesh. The map is contoured at 2.5 σ. (**b**) LBQ657 (yellow) makes H-bonding interactions (black dotted lines) with residues of NEP (white carbon atoms) and coordinates with zinc (pink). The active site surface of NEP is shown in grey.

The extracellular domain of human NEP (residues 54–749) was expressed using the baculovirus system. NEP in complex with LBQ657 crystallized in the orthorhombic space group P2_1_2_1_2_1_ with two copies in the asymmetric unit diffracting to 2.0 Å resolution. The structure was solved by molecular replacement. The initial difference electron density clearly represents LBQ657 (Fig. 2a). As reported previously^7^, the extracellular domain of NEP is comprised of two largely α-helical subdomains, with domain 1 predominantly containing C-terminal residues while domain 2 is formed mainly from the N-terminal half of the protein. The two domains are arranged to harbor a large cavity in the center that contains the catalytic machinery which is formed by residues from domain 1 (Fig. 2b). LBQ657 is bound to the active site of NEP by an intricate network of interactions that involves all functional groups of the compound giving rise to the high inhibitory potency of 5 nM^12^. In the crystal structure, the catalytic zinc atom of NEP is ligated by the side chains of residues His583 (2.0 Å), His587 (1.9 Å) and Glu646 (2.0 Å) with the fourth coordination provided by the carboxylate oxygen adjacent to the P1 methyl of the compound (2.0 Å). The second oxygen atom of this carboxylate is at a distance of 2.7 Å from the catalytic zinc. The methyl group of LBQ657 is pointing towards the shallow S1 pocket making hydrophobic interactions with Phe544. There are two chiral centers in LBQ657 with the specific configuration in both centers facilitating optimal interactions with the active site of NEP. In fact, the other three diastereoisomers of LBQ657 reported by Ksander *et al*. show a reduction in the potency by about 5 to more than 100-fold^12^.

**Figure 3:**
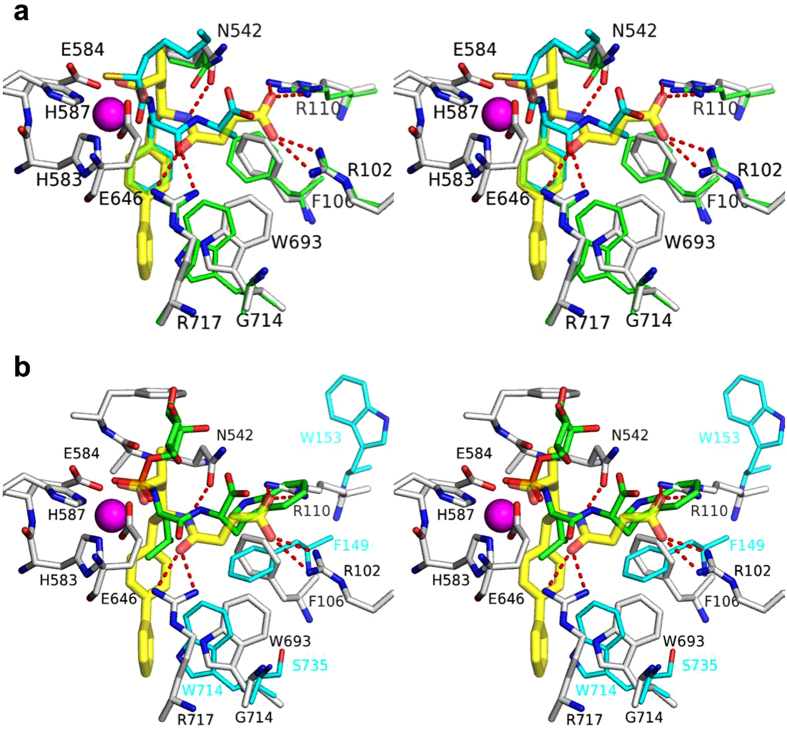
Superimposed active site residues of the NEP:LBQ657 complex (white and yellow carbon atoms) with **(a)** NEP in complex with a compound containing a benzyl in P1’ (1R1I^8^; cyan and green carbon atoms) and **(b)** ECE-1^13^ in complex with phosphoramidon (green and cyan carbon atoms).

The backbone amide of LBQ657 forms H-bonding interactions with the side chains of Asn542 and Arg717. The amide directionality in LBQ657 is different relative to peptide substrate and inhibitors reported in published crystal structures^7^,^8^,^9^,^10^,^11^. In LBQ657, the –NH of the amide is attached to the chiral carbon atom, while in the inhibitors from crystal structures reported previously^7^,^8^,^9^,^10^,^11^, the attachment occurs via the amide carbonyl. It is interesting to note that for both types of amide, the nitrogen and oxygen atoms occupy similar positions in the complex structures and are engaged in the same type of interactions (Fig. 3a). The succinic acid in P2’ has been shown to be optimal compared to analogs with shorter or extended chain length^12^. It places the carboxylic acid in an ideal position for optimal interaction with Arg102 and Arg110, unique for LBQ657.

The P1’ biphenyl of LBQ657 binds deeply in the S1’ subsite of NEP, inducing conformational changes for optimal hydrophobic interactions. A concerted change in conformation of residues Trp693 and Phe106 when a compound containing a biphenyl group is bound in P1’ has been reported previously^8^. When comparing our co-crystal structure to one with a compound harboring a benzyl in P1’^8^, an analogous induced fit of Trp693 towards the S2’ subsite is observed, which in turn leads to a conformational change of Phe106 (Fig. 3a). In NEP the space occupied by the new orientation of Trp693 is available because the adjacent Gly714 does not project a sidechain towards this area. LBQ657 does not inhibit angiotensin converting enzyme (ACE), or endopeptidases homologous to NEP such as ECE-1 (39% sequence identity with NEP) and NEP2 (55% sequence identity with NEP) when tested at a maximum concentration of 30 μM (unpublished data). The residues corresponding to Gly714 are Ser735 (ECE-1)^13^ and Leu744 (NEP2) and their side chains will impose steric constraints to the conformational change of Trp714 (ECE-1) and Trp723 (NEP2), respectively (Fig. 3b). The X-ray structure of ECE-1 confirms Ser735 at the position corresponding to NEP Gly714 (Fig. 3b)^10^. Additional active site residue differences to NEP will also have an impact on selectivity, such as Arg110_NEP_ →Trp153_ECE-1_.

All of the molecular interactions between LBQ657 and the enzyme are non-covalent, in line with a reversible inhibition mode. The crystal structure in addition shows the presence of a sub-pocket extending from the meta-position of the second phenyl ring in P1’ to the backbone carbonyl of Arg717.

In conclusion, the reported crystal structure shows how LBQ657 binds to NEP, highlighting the interactions in the catalytic site. The crystal structure unravels the importance of the chiral centers of LBQ657 for optimal binding to NEP, zinc ligation by the carboxylate oxygen adjacent to the P1 methyl, and conservation of hydrogen bonding interactions by the backbone amide. An induced fit binding of the bulky biphenyl group in the S1’ subsite sheds light on the plasticity of NEP and yields insights into the mechanism of selectivity. Further work probing the interactions observed in this co-crystal structure, especially the structure activity relationship around the second phenyl ring will be reported separately.

## Methods

### Protein expression

Human Neprilysin (residues Gly52-Trp749) was expressed using the baculovirus expression system, utilizing a Cathepsin D leader sequence and C-terminal Strep tag. A 10 liter culture of SF21cells was infected at a multiplicity of infection (MOI) of 1.0 and the supernatant harvested after 72 h and frozen at −80 °C.

### Protein purification

Four liters of baculovirus expression supernatant were allowed to thaw overnight and any precipitate that had formed was removed by centrifugation at 10,000 g for 15 min. The supernatant, following addition of a 1/10 volume of 10× stock buffer A (10 mM Tris at pH 8, 250 mM NaCl, 1 mM MgCl_2_ and 1 mM CaCl_2_), was loaded onto 60 ml column of Con A Sepharose equilibrated with buffer A. After loading the sample, the column was washed with buffer A until the UV absorbance returned to baseline. The column was then eluted directly using a buffer B (buffer A, containing 0.5 M mannopyrannoside and 0.5 M glycopyrannoside). The NEP-containing fractions (450 ml) were dialyzed over the weekend at 4 °C against 25 L of 25 mM Tris at pH 8.0. 350 ml of 3.6 M ammonium sulfate was added to 500 ml of the retentate and following centrifugation (10,000 g; 5 min), the supernatant was loaded onto a 22 ml column of Source™ Phenyl resin which had been equilibrated with buffer C (50 mM Tris, 1.5 M ammonium sulfate). The column was eluted with a gradient of 0–50% buffer D (buffer C without ammonium sulfate) over 30 column volumes with a final step to 100% buffer D. The eluted protein peak was dialyzed against 25 mM Tris at pH 8, containing 1 mM MgCl_2_ (buffer E) and further diluted with 2 volumes of the same buffer prior to loading onto a 1 ml MonoQ™ anion-exchange column. The column was eluted with a 0–25% gradient of buffer F (buffer E plus 1 M NaCl) over 18 column volumes. The eluted protein peak was pooled with care being taken to exclude small shoulders on the trailing edge of the peak. The pool was concentrated to 15.1 mg/ml using a 5 kDa cut-off centrifugal concentrator.

### LBQ657 stock preparation

A stock solution at 100 mM concentration in 100% DMSO was prepared using powder of LBQ657.

### Crystallization

For crystallization, the protein was used at a concentration of 10 mg/ml in 20 mM Tris at pH 8.0, 125 mM NaCl, 2 mM MgCl_2_. LBQ657 (100 mM in DMSO) was mixed with the protein to a final inhibitor concentration of 1mM. Crystallization experiments were performed at room temperature using the hanging drop vapor diffusion method with a 1:1 ratio of inhibitor protein complex mixed with the reservoir solution containing 25% (w/v) PEG3350, 200 mM ammonium acetate, 100 mM BisTris, pH 6.5. Crystals appeared overnight. Before data collection, the crystals were transferred to 25% PEG3350, 200 mM ammonium acetate, 100 mM BisTris at pH 6.5, 20% (v/v) glycerol and flash frozen in liquid nitrogen.

### Data collection, structure determination and refinement

X-ray diffraction data were collected at the PXII beamline of the Swiss Light Source (Villigen, Switzerland) at a wavelength of 1.00 Å at 100 K and processed with XDS as implemented in the program package APRV^14^,^15^. The crystals diffracted to 2.0 Å resolution and belong to the space group P2_1_2_1_2_1_ with two monomers in the asymmetric unit. The structure was solved by molecular replacement using the coordinates of PDB entry 1DMT as a search model^7^. Iterative cycles of model building and refinement were carried out using programs COOT^16^, Refmac^17^ and BUSTER^18^. The final R-factor is 19.3% (R_free_ 22.8%). The data collection and refinement statistics are summarized in Table 1. The coordinates of the refined structure have been deposited in the Protein Data Bank (5jmy).

## Additional Information

**How to cite this article**: Schiering, N. *et al*. Structure of neprilysin in complex with the active metabolite of sacubitril. *Sci. Rep.* **6**, 27909; doi: 10.1038/srep27909 (2016).

## Figures and Tables

**Figure 1:**
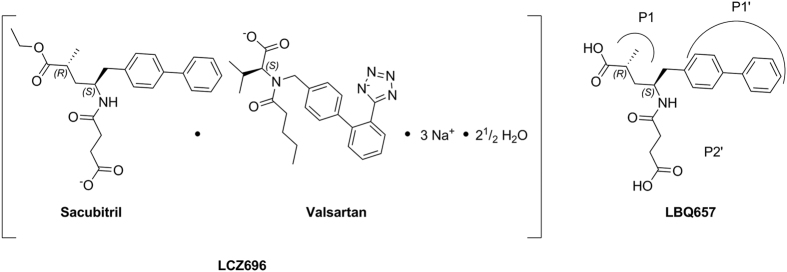
Chemical structures of LCZ696, sacubitril, valsartan and LBQ657.

**Table 1:** Data collection and refinement statistics (molecular replacement).

Data collection	
Space group	P2_1_2_1_2_1_
Cell dimensions	
*a*, *b*, *c* (Å)	59.7, 109.1, 248.0
Resolution (Å)	65.9 − 2.0 (2.06 − 2.00)
*R*_sym_	0.065 (0.493)
*I*/σ*I*	13.8 (2.6)
Completeness (%)	98.2 (97.8)
Redundancy	4.0 (3.8)
Refinement	
Resolution (Å)	29.28 − 2.00
No. reflections	108529
*R*_work_/*R*_free_	0.193/0.228
No. atoms	
Protein	11190
Ligand/ion	170
Water	518
*B*-factors	
Protein	42.4
Ligand/ion	26.0
Water	45.6
R.m.s. deviations	
Bond lengths (Å)	0.01
Bond angles (°)	1.06

*Values in parentheses are for highest-resolution shell.
